# Hydrophilic Polyelectrolyte Multilayers Improve the ELISA System: Antibody Enrichment and Blocking Free

**DOI:** 10.3390/polym9020051

**Published:** 2017-02-12

**Authors:** Xing Lai, Gan Gao, Junji Watanabe, Huiyu Liu, Heyun Shen

**Affiliations:** 1 Beijing Key Laboratory of Bioprocess, Beijing Advanced Innovation Center for Soft Matter Science and Engineering, Beijing Laboratory of Biomedical Materials, College of Life Science and Technology, Beijing University of Chemical Technology, Beijing 100029, China; lx438687459@163.com (X.L.); 2016201167@mail.buct.edu.cn (G.G.); 2 Faculty of Science and Engineering, Konan University, 8-9-1 Okamoto, Higashinada, Kobe 658-8501, Japan; junjiknd@konan-u.ac.jp

**Keywords:** polyelectrolyte multilayers, ELISA, poly(acrylic acid), hydrophilic surface

## Abstract

In this study, polyelectrolyte multilayers were fabricated on a polystyrene (PS) plate using a Layer-by-Layer (LbL) self-assembly technique. The resulting functional platform showed improved performance compared with conventional enzyme-linked immunosorbent assay (ELISA) systems. Poly(diallyldimethylammonium chloride) (PDDA) and poly(acrylic acid) (PAA) were used as cationic and anionic polyelectrolytes. On the negatively-charged (PDDA/PAA)_3_ polyelectrolyte multilayers the hydrophilic PAA surface could efficiently decrease the magnitude of the noise signal, by inhibiting nonspecific adsorption even without blocking reagent adsorption. Moreover, the (PDDA/PAA)_3_ substrate covalently immobilized the primary antibody, greatly increasing the amount of primary antibody adsorption and enhancing the specific detection signal compared with a conventional PS plate. The calibration curve of the (PDDA/PAA)_3_ substrate showed a wide linear range, for concentrations from 0.033 to 33 nM, a large specific signal change, and a detection limit of 33 pM, even though the conventional blocking reagent adsorption step was omitted. The (PDDA/PAA)_3_ substrate provided a high-performance ELISA system with a simple fabrication process and high sensitivity; the system presented here shows potential for a variety of immunosensor applications.

## 1. Introduction

The enzyme-linked immunosorbent assay (ELISA) system is extensively applied to perform immunoassays. The ELISA system combines specific antibodies with sensitive and simple enzyme assays, by using antibodies or antigens coupled to an easily-assayed enzyme [1]. Owing to its specificity, excellent linearity and high precision, the ELISA system is widely used in a variety of fields, including protein detection and quantification, detection of sulfonamide residues in food, and clinical medicine [2,3,4]. However, current ELISA techniques have some disadvantages, including low primary antibody adsorption, poor blocking efficiency, and a requirement for complicated, multiple steps [5]. The conventional ELISA system on a PS plate suffers from relatively poor sensitivity, a low surface-to-volume ratio, and random orientation of the adsorbed antibody or antigen, which is not optimal for subsequent antibody–antigen recognition and binding [6]. It is, therefore, crucial to develop new methods with a higher sensitivity and a lower limit of detection (LOD) for routine diagnosis [7,8].

Many researchers have devoted much effort to create new devices to improve the sensitivity of the ELISA system [9,10,11,12,13,14,15]. Nagasaki et al. immobilized poly(ethylene glycol) (PEG) with antibodies or aptamers on a substrate, to evaluate an immunoassay system, which not only enhanced the corresponding epitope and improved the protein-aptamer interaction, but also efficiently suppressed nonspecific adsorption [11,12]. Watanabe et al. designed a fluorescence resonance energy transfer (FRET) system on the surface of phosphorylcholine (PC) group-enriched nanoparticles for rapid immunoassay performance [13]. In this system the immunoassay protocol was quite simple, involving only the mixing of FRET-installed nanoparticles and antigen molecules; nonspecific protein adsorption was effectively suppressed by the hydrophilic nanoparticles. Serizawa et al. confirmed that macromolecularly double-stranded regular nanostructures of ultrathin poly(methyl methacrylate) (PMMA) stereocomplex films could efficiently enhance the antibody-antigen recognization [14]. Jia et al. increased the detection sensitivity using antibody-modified gold nanoparticles (GNP), and antibody-modified magnetic nanoparticles [15]. The sensitivity of the ELISA system can be improved via changes to each step of protein adsorption and/or reaction in the conventional ELISA (where those steps include primary antibody immobilization, blocking reagent adsorption, and antigen and secondary antibody reaction). It is known that the properties of the substrate surface are a critical factor in controlling protein adsorption in the ELISA system; hence, surface modification could be the most effective method for improving the conventional ELISA system.

Polyelectrolyte multilayers (PEMs) provide an effective and simple method for surface modification, and have been applied for a variety of functions in different fields since they were developed by Decher in the 1990s [16]. The surface properties of PEMs can be controlled at the nanometer level using Layer-by-Layer (LbL) techniques, in which polyelectrolytes with opposite charges are deposited on the surfaces [17]. PEMs have been used in many studies to modify the surface of biological materials and to control protein adsorption behavior [18,19,20,21,22,23,24]; for example, PEMs have been used for single cell analysis, have been applied as biocompatible coatings to improve the immobilization between tissues and implants, and have been used to create a protein-resistant surface comprising poly(acrylic acid) (PAA) homopolymer multilayers. PEMs have not been applied only as films; PEM capsules play a role in a variety of cancer detection and treatment methods [25]. Electrostatic forces, hydrogen bonding, and hydrophobic interactions are considered to be the driving forces responsible for protein adsorption on PEMs. We fabricated differently-charged PEMs on each side of a substrate to produce different amounts of protein adsorption on each side [26]. As a result of these experiments, we considered that PEMs could be used to control the protein adsorption in each step of the ELISA system, leading to improvement of the immunoassay performance. We have demonstrated that cationic PEM, poly(diallyldimethylammonium chloride) (PDDA)/poly(sodium 4-styrenesulfonate) (PSS) shows excellent sensitivity compared with standard polystyrene (PS) plates due to that of high surface coverage of the blocking reagent (100%) [27]. Indirect ELISAs typically lack the ability to selectively bind a specific target. A sandwich immunoassay system is usually superior for specific samples [28].

In this study, it was found that hydrophilic PEMs effectively improved the sensitivity of the ELISA system, compared with a conventional PS plate. The cationic and anionic polyelectrolytes used were poly(diallyldimethylammonium chloride) (PDDA) and PAA, respectively. The hydrophilic properties of PAA not only suppressed nonspecific protein adsorption, decreasing the magnitude of the noise signal even in the absence of a blocking reagent adsorption step, but also increased primary antibody adsorption on the substrate via covalent immobilization, thus increasing the specific detection signal. The hydrophilic PEMs in the ELISA system, therefore, not only increased the signal-to-noise ratio (S/N) to enhance the detection sensitivity, but also, more importantly, simplified the blocking process of the conventional ELISA system to create a more convenient immunoassay process.

## 2. Materials and Methods

### 2.1. Materials

Poly(diallyldimethylammonium chloride) (PDDA, No. 17338; Polyscience Inc., Niles, IL, USA; *M*_w_ = 2.4 × 10^5^ g/mol) is a strong polyelectrolyte with a positive charge, and poly(acrylic acid) (PAA, No. 181285; Sigma, St. Louis, MO, USA; *M*_w_ = 4.5 × 10^5^ g/mol) is a weak polyelectrolyte with a negative charge. Rabbit antimouse IgG (M7023), mouse IgG (I5381), goat anti-mouse IgG-horseradish peroxidase conjugate (goat antimouse IgG-HRP; A4416), ovalbumin from chicken egg white (OVA, A5503), and *N*-hydroxysuccinimide (NHS) were acquired from Sigma (St. Louis, MO, USA). 1-ethyl-3-(3-dimethylaminopropyl) carbodiimide hydrochloride (EDC) was purchased from Dojindo Co. Ltd., Shanghai, China. Disodium hydrogen phosphate and monometallic sodium orthophosphate were purchased from Beijing Chemical Reagent Co. Ltd. (Beijing, China). Tris(hydroxymethyl) aminomethane hydrochloride (Tris-HCl; Acros Organics, Fair Lawn, NJ, USA) was used in the preparation of a buffer solution. 96 wells PS microplate (Costar 3599) was purchased from Corning Incorporated (Corning, NY, USA). The chemicals were used without further purification. Ultrapure water was used throughout the experiments.

### 2.2. Fabrication of PEMs in PS Microplate Wells

In this study, the polyelectrolyte PDDA and PAA were separately dissolved in 50 mmol/L tris-HCl (pH 7.4), and the concentration of the solutions was 0.2 mg/mL. The ionic strength of the solutions was then adjusted to 0.15 mol/L, using NaCl. It was necessary to adjust the ionic strength to facilitate the complete adsorption of each polyelectrolyte. First, 300 or 120 μL of the PDDA aqueous solution was dropped into the wells of a PS microplate (48 or 96 wells) and left for 1 min at room temperature, and the plate was then rinsed twice with ultrapure water for 20 s (and the rinsing water was then removed). Next, the PAA aqueous solution was added in the same way. This alternating drop-coating process was repeated to produce the required number of polyelectrolyte layers.

### 2.3. Determination of Physical Adsorption and Covalent Immobilization of Protein

The primary antibody (rabbit anti-mouse IgG; 30 μg/mL in 0.03 mol/L phosphate-buffered saline (PBS); pH 7.4) and OVA (1 mg/mL; PBS, 0.03 mol/L; pH 7.4) were adsorbed onto the PEMs (48 wells) at room temperature for 2 h. Later, the substrates were rinsed three times with ultrapure water for 20 s, and then with 1 wt % of *n*-sodium dodecyl sulfate (SDS; Wako Pure Chemical Industries, Ltd., Osaka, Japan) for 30 min to remove the adsorbed proteins. The protein concentration was measured at 570 nm using a Micro BCA kit (Pierce, No. 23235, Rockford, IL, USA), using a multi-well plate reader (PT-3502G, Beijing Potenov Technology Co. Ltd., Beijing, China). We also investigated the effects of changing the antibody concentration on the amount of physical adsorption of antibody. The results showed that the amount of antibody adsorption did not change with higher antibody concentrations (Figure S1).

To increase the amount of primary antibody adsorption, we used a chemical method to immobilize the primary antibody on the (PDDA/PAA)_3_ (six-step assembly; the outermost layer was PAA). After the formation of (PDDA/PAA)_3_ in the 48-well plate, EDC (1 mg/mL, 150 μL/well) and NHS (1 mg/mL, 150 μL/well) at 0.03 M PBS (pH = 6.33) were added at room temperature for 20 min. After rinsing with PBS (0.03 M, pH = 7.4) twice, the primary antibody (100 μL/well) was added to the wells at room temperature, and left for 2 h. The antibody concentration was optimized for covalent immobilization. The amount of unreacted antibody in the supernatant was measured using a Micro BCA kit, to determine the amount of covalently-immobilized antibody.

### 2.4. ELISA Protocol on the PS and PEMs Substrate

The primary antibody (100 μL/well) was adsorbed on the PS and PEMs substrate (96-well) via physical adsorption and chemical immobilization, as described above. Next, OVA (1 mg/mL; 200 μL/well) was adsorbed on each well at 37 °C for 1 h. The antigen and secondary antibody were mouse IgG (100 μL/well; PBS 0.03 mol/L with 0.1 wt % OVA; pH 7.4) and goat antimouse IgG-HRP, respectively, and their reaction processes were carried out at 37 °C for 1 h. The concentration of antigen ranged from 0.0134 to 33.5 nM. The optimal concentration of secondary antibody was 1.7, 3.4, and 1.7 nM on the PS plate, (PDDA/PAA)_3_, and (PDDA/PAA)_3_PDDA (seven-step assembly; the outermost layer was PDDA), respectively (Figure S2). In the absence of OVA adsorption, the optimal concentration of secondary antibody was 1.7 nM on the PS plate and the PEMs. Tetramethylbenzidine (34028, one-step ultra TMB-ELISA, Thermo Fisher Scientific Co. Ltd., Rockford, IL, USA) was added to each well for the HRP reaction, then the absorbance of the solution was then measured at 450 nm using the multi-well plate reader. A brief protocol for the conventional ELISA process is set out in the supporting information.

## 3. Results and Discussion

### 3.1. Determination of Primary Antibody and Blocking Adsorption

PEMs can be deposited on a substrate of any morphology using a simple alternating dip-coating process, and PEMs hold significant advantages for the effective control of protein adsorption and cell adhesion [21]. Several groups demonstrated the PAA composite materials can resist various protein adsorption due to the hydrophilic carboxyl group on the surface [29,30,31]. Based on the above analysis, we hypothesized that PDDA/PAA PEMs could be used to control the protein adsorption in each step of the ELISA method, leading to improvements in the sensitivity of immunoassays.

**Figure 1:**
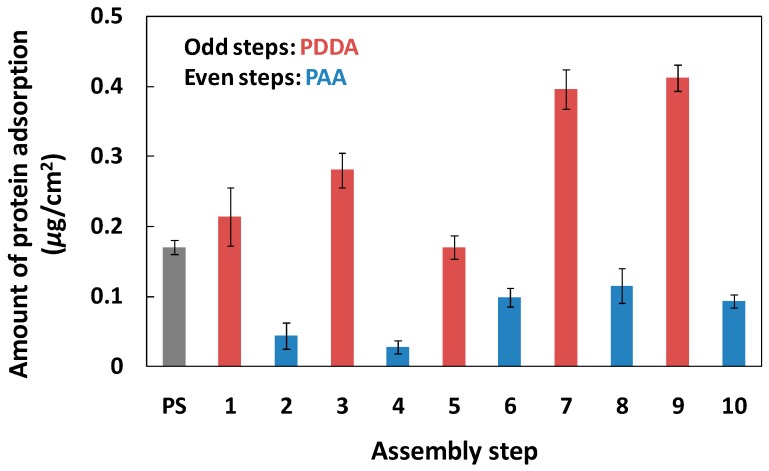
Amount of primary antibody adsorption on the PS plate and the PDDA/PAA PEMs. The odd and even steps represent the PDDA layers and PAA layers, respectively (*n* = 3).

**Figure 2:**
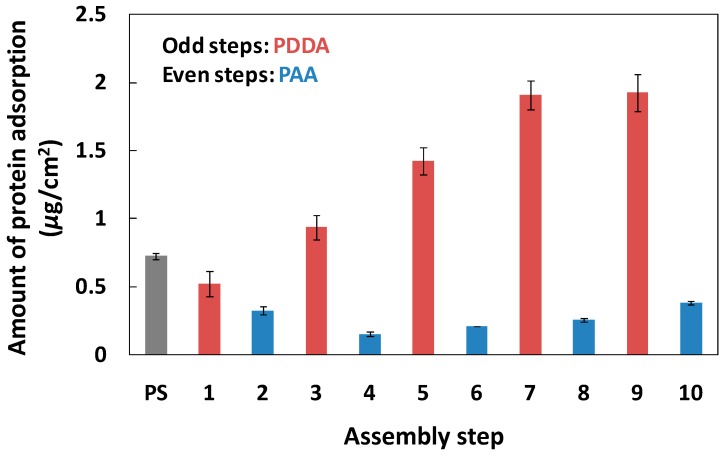
Amount of OVA adsorption on the PS plate and the PDDA/PAA PEMs. The odd and even steps represent the PDDA layers and PAA layers, respectively (*n* = 3).

We measured the PDDA/PAA PEM formation using quartz crystal microbalance (QCM) system. The condition for PEM formation was same as on PS plate. From the change of QCM frequency shift, we cleared that the membrane thickness gradually increased with increase of assembly step (Figure S3). We investigated the primary antibody (Figure 1) and blocking reagent (OVA) (Figure 2) adsorption on a PS plate, and on PDDA/PAA PEMs with one to ten layers. The amount of IgG adsorbed on the positively-charged PEMs (i.e., with the outermost layer being PDDA) was 0.21–0.41 μg/cm^2^, and on the negatively-charged PEMs (i.e., with the outermost layer being PAA) and PS plate the adsorbed amounts were 0.03–0.12 and 0.17 μg/cm^2^, respectively. The adsorption capacity of the PAA layers was much lower than that of the PDDA layers and the PS plate, and the maximum amount of IgG adsorption was obtained on the ninth layer. The isoelectric point (pI) of IgG is 6.4–9.0; hence, it exhibited a nearly neutral charge in the buffer solution in this study. Therefore, the main driving force for protein adsorption on the PEMs were not electrostatic interactions between the protein and the PEMs interface, but a hydrophobic/hydrophilic interaction; in particular, the hydrophilic interface provided by the PAA layers induced efficient inhibition of the adsorption of IgG. These results suggested that the PAA layers also can reduce nonspecific protein adsorption from the secondary antibody, decreasing the magnitude of the noise signal.

As shown in Figure 2, the amount of OVA adsorbed on the PAA layer of the PEMs was 0.15–0.38 μg/cm^2^, much lower than the amount adsorbed on the PDDA layer of the PEMs (0.52–1.93 μg/cm^2^) and the PS plate (0.72 μg/cm^2^). The pI of OVA is 4.6, so the OVA was negatively charged in the buffer solution (pH 7.4). The adsorption of OVA on the PS plate mainly depended on hydrophobic interactions, but the PEMs precisely regulated the quantity of OVA adsorption via electrostatic interactions between the protein and the PEMs [27]. Therefore, the blocking effect of OVA on the PAA layer of the PEMs was reduced, compared with the PDDA layer of the PEMs, because of the lower coverage rate of OVA on the PAA layer of the PEMs (Table 1).

Under normal circumstances, when PEMs have six or more layers, the characteristics of the membrane surface are exactly determined by the outermost polyelectrolyte layer, because the uniformity of PEMs can be highly controlled [32]. Moreover, the amounts of protein adsorption (IgG and OVA) were almost equal on the same charged PEMs over 6–10 layers (Figure 1 and Figure 2). Therefore, we selected negatively-charged six-step and positively-charged seven-step PEMs, using (PDDA/PAA)_3_ and (PDDA/PAA)_3_PDDA, to investigate the characteristics of the PEMs in the ELISA system. Table 1 summarizes the amount of IgG and OVA adsorption and their surface coverage on the PS plate, and on the (PDDA/PAA)_3_ and (PDDA/PAA)_3_PDDA PEMs. Although both were negatively charge, compared with the (PDDA/PSS)_3_ PEMs [27], (PDDA/PAA)_3_ not only showed lower amounts of adsorbed OVA, but the hydrophilic surface of PAA also inhibited primary antibody adsorption. These results suggested that the (PDDA/PAA)_3_ substrate played a role as a blocking surface to inhibit nonspecific antigen and secondary antibody adsorption, and that the step of blocking reagent adsorption could be omitted to simplify the process used with conventional ELISA systems.

### 3.2. Blocking Ability of PAA PEMs

**Figure 3:**
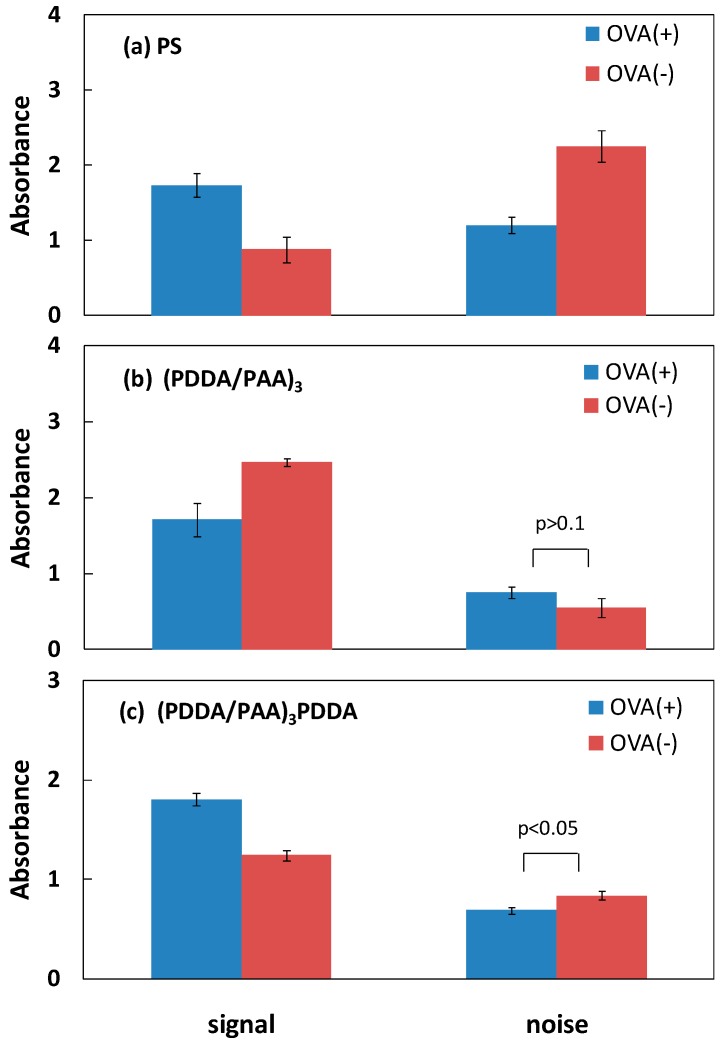
Specific signal and noise associated with antigen detection, with and without OVA blocking reagent adsorption, on the PS plate (**a**); (PDDA/PAA)_3_ (**b**); and (PDDA/PAA)_3_PDDA (**c**) (*n* = 3).

**Figure 4:**
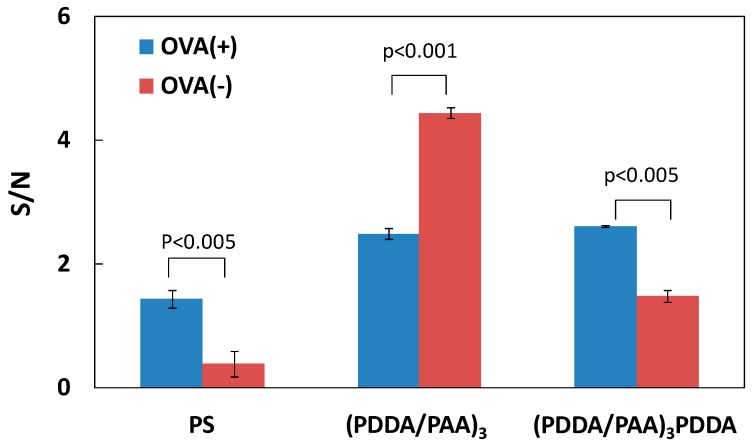
S/N ratio for antigen detection with, and without, OVA adsorption on the PS plate, (PDDA/PAA)_3_, and (PDDA/PAA)_3_PDDA (*n* = 3).

One aim of this work was to validate the hypothesis that the hydrophilic nature of the PAA PEM-modified PS plate can directly decrease nonspecific protein adsorption without any blocking of reagent adsorption, leading to a simple and sensitive ELISA system. In a conventional ELISA system, albumin protein is typically used to block the solid substrate to reduce nonspecific protein (antigen and/or secondary antibody) adsorption. Hence, we designed contrast experiments using the conventional ELISA system on the PS plate, and differently charged PEMs, with and without an OVA blocking step in each case. Figure 3 shows the specific signal and noise associated with antigen detection on the PS plate, and the (PDDA/PAA)_3_ and (PDDA/PAA)_3_PDDA PEMs, with and without OVA adsorption. The term “specific signal” denotes the signal resulting from the subtraction of the noise from the antigen-secondary antibody reaction signal; in this case the noise was the nonspecific secondary antibody adsorption signal. As shown in Figure 3a, on the conventional PS plate, the noise value obtained without OVA adsorption was approximately two times greater than that obtained with OVA adsorption, resulting in a much lower specific signal, which even was half the magnitude of the noise. In contrast, on the (PDDA/PAA)_3_ substrate, the noise was not notably different with and without OVA adsorption, and the specific signal without OVA adsorption was higher than that obtained with OVA adsorption (Figure 3b). This was likely because nonspecific protein adsorption was efficiently inhibited by the hydrophilic (PDDA/PAA)_3_ surface itself. However, for the (PDDA/PAA)_3_PDDA substrate, the specific signal was reduced and the noise was greater in the absence of OVA adsorption, similar to the PS plate (Figure 3c). Further, we compared the specific signal-to-noise ratio (S/N) obtained with and without OVA adsorption on the different substrates (Figure 4). On the (PDDA/PAA)_3_ substrate, the S/N obtained without OVA adsorption was two times greater than that obtained with OVA adsorption. Conversely, the S/N obtained without OVA adsorption was reduced on both the PS plate and the (PDDA/PAA)_3_PDDA substrate. Moreover, the S/N obtained for (PDDA/PAA)_3_ without OVA (4.44) was approximately three times and two times greater than that obtained for the PS plate (1.44) and (PDDA/PAA)_3_PDDA (2.61) with OVA adsorption, respectively. These results confirmed that the (PDDA/PAA)_3_ substrate provided a sensitive ELISA system without the need for any blocking process, simplifying and improving upon the conventional PS plate system.

**Figure 5:**
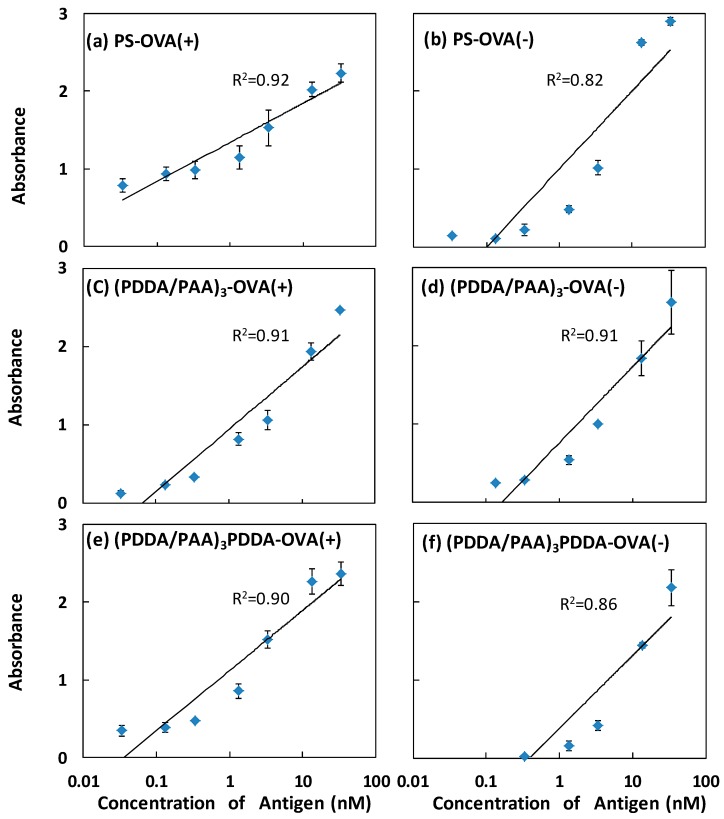
Specific signal as a function of antigen concentration on the PS plate (**a**,**b**); and the (PDDA/PAA)_3_ (**c**,**d**); and (PDDA/PAA)_3_PDDA substrates (**e**,**f**); with (**a**,**c**,**e**); and without (**b**,**d**,**f**) OVA adsorption (*n* = 3).

Figure 5 shows the specific signal as a function of antigen concentration from 0.0134 to 33.5 nM, with, and without, OVA adsorption, on the different substrates. The (PDDA/PAA)_3_ substrate showed a wide detection range, a large correlation coefficient (*R*^2^), and a large signal change over the full concentration range both with, and without, OVA adsorption; these results demonstrated that the sensitive detection capacity was available regardless of the presence of OVA. In contrast, on the PS plate and the (PDDA/PAA)_3_PDDA substrate, the effective concentration detection range without OVA adsorption was narrow, and the linearity of the calibration curve was poor compared with that obtained with OVA adsorption, resulting in a loss in detection sensitivity. The *R*^2^ value for the calibration curve was consistent for the cases with, and without, OVA adsorption only on the (PDDA/PAA)_3_ substrate. The fundamental reason for this was that the hydrophilic nature of the PAA led to the inhibition of the nonspecific adsorption by the (PDDA/PAA)_3_ substrate; hence, even if the OVA adsorption step was omitted, the same sensitive detection limit was achieved. The (PDDA/PAA)_3_ substrate reduced the number of steps required, compared with the conventional ELISA system, as a result of the effective nonspecific protein adsorption blocking abilities of the (PDDA/PAA)_3_ substrate.

### 3.3. Higher Sensitivity Induced by Antibody Enrichment on (PDDA/PAA)_3_

As shown previously, the amount of primary antibody adsorbed on the (PDDA/PAA)_3_ via physical adsorption was only 0.1 μg/cm^2^, but a higher degree of primary antibody immobilization provide more antigen binding sites and increase the specific signal; this is one of the important factors responsible for the improvement of the ELISA sensitivity. To increase the amount of primary antibody, we used a covalent method to immobilize the primary antibody on the (PDDA/PAA)_3_ substrate through the carboxyl groups of the PAA. The carboxyl groups on the PAA surface were activated using EDC, and the coupling efficiency was improved using NHS. It was considered that a higher initial concentration of the primary antibody could also increase the degree of primary antibody immobilization; hence, we regulated different antibody concentrations of 10, 20, and 30 μg/mL. Table 2 shows that, as expected, the amount of primary antibody immobilization increased along with the initial primary antibody concentration. Moreover, the amount of covalent immobilization obtained with a concentration of 10, 20, and 30 μg/mL, was 2.8, 4.2, and 5.6 times higher than the physically adsorbed amount (0.1 μg/cm^2^), respectively.

**Figure 6:**
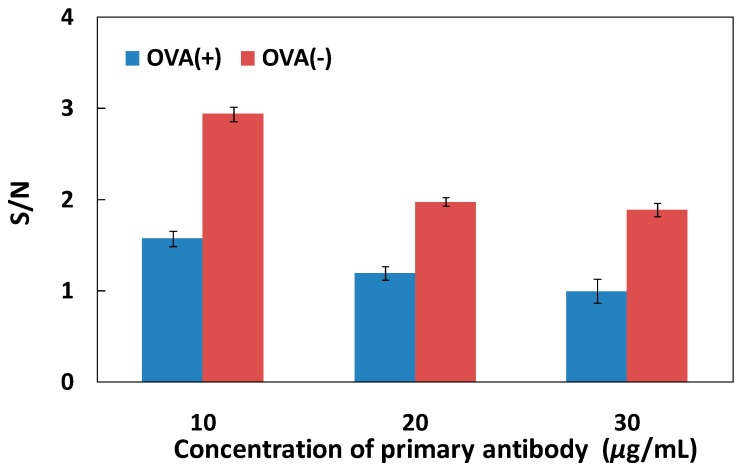
S/N ratio for antigen detection with different primary antibody concentrations, for covalent immobilization on the (PDDA/PAA)_3_ substrate with, and without, OVA adsorption (*n* = 3).

To obtain the most sensitive ELISA system on the (PDDA/PAA)_3_ substrate, we also investigated the S/N ratio for antigen detection in different primary antibody concentrations for covalent immobilization. As shown in Figure 6, the S/N ratio increased gradually with decreases in the primary antibody concentration, with and without OVA adsorption, and the maximum S/N value was achieved at a concentration of 10 μg/mL, despite the fact that the amount of antibody immobilized at 20 and 30 μg/mL was greater than at 10 μg/mL (Table 2). We hypothesized that when the antibody concentration was 20 or 30 μg/mL, the effective binding sites of the primary antibody were covered, due to the presence of multiple layers of immobilized antibody; hence, the detection molecules could not be efficiently captured, inducing a decrease in the specific signal and the S/N ratio. With high concentrations of the immobilized antibody, the carboxyl group content decreased with increases in the amount of the immobilized primary antibody, resulting in a reduction of the ability to inhibit nonspecific adsorption; thus, the S/N value decreased. A concentration of 10 μg/mL was, therefore, the optimal concentration for covalent immobilization, not only increasing the number of effective primary antibody binding sites compared with physical adsorption, but also providing a relatively hydrophilic surface (i.e., the residual carboxyl groups), achieving an appropriate balance between specific signal enhancement and noise reduction. The S/N value in the ELISA system without OVA was higher than that of the ELISA system with any added concentration of OVA.

**Figure 7:**
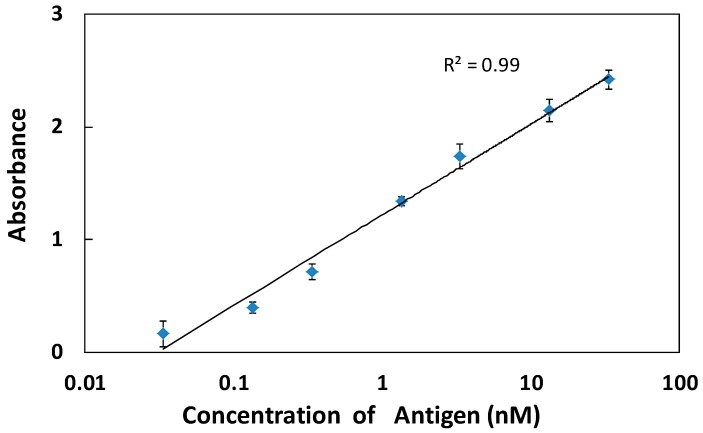
Specific signal as a function of antigen concentration on the (PDDA/PAA)_3_ substrate, without OVA adsorption. The primary antibody was covalently immobilized on the substrate at a concentration of 10 μg/mL (*n* = 3).

Since the hydrophilic properties of PAA enhanced the S/N ratio by increasing the antibody quantity and decreasing the noise, we investigated the conventional ELISA system with no blocking step on the (PDDA/PAA)_3_ substrate. Figure 7 shows the specific signals obtained in different antigen concentrations (from 0.0134 to 33.5 nM) on the (PDDA/PAA)_3_. The primary antibody was covalently immobilized on the substrate at a concentration of 10 μg/mL. Compared with the results obtained using physical adsorption of antibody on the (PDDA/PAA)_3_ substrate (Figure 5c,d), the linearity of the calibration curve was excellent (*R*^2^ = 0.99), with smaller standard deviations; the effective detection range was large, with an limit of detection (LOD) of 13.4 pM. More importantly, compared with the conventional PS plate system (Figure 5a), not only was the *R*^2^ value high, but the slope of the calibration curve was also large; that is, the change in the specific signal was large, with high sensitivity, even though the step of blocking reagent adsorption on the (PDDA/PAA)_3_ substrate was omitted. The hydrophilic (PDDA/PAA)_3_, therefore, provided a more sensitive detection system, compared with the conventional ELISA system, because the specific signal was enhanced via the covalent immobilization of primary antibody, and the magnitude of the nonspecific signal (noise) was reduced by the inhibition of nonspecific adsorption by the extremely hydrophilic surface. The number of detection steps was reduced by omitting the reagent adsorption blocking step, thus improving over the conventional ELISA system.

**Figure 8:**
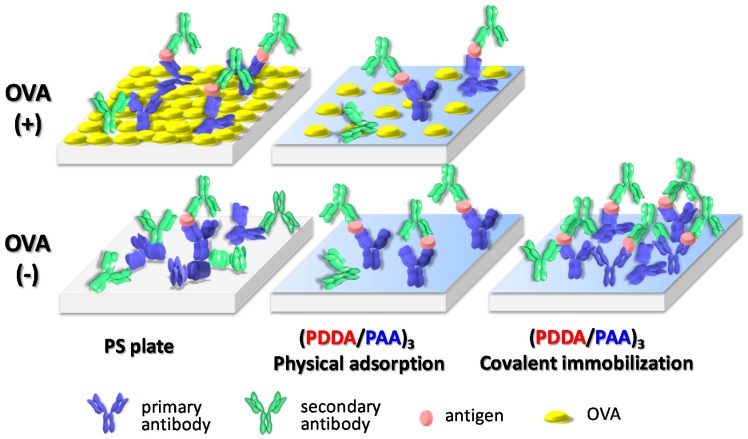
Proposed mechanism responsible for the signal and noise associated with secondary antibodies, on the PS plate and (PDDA/PAA)_3_, with, and without, OVA adsorption. The primary antibody could be immobilized on the (PDDA/PAA)_3_ via physical adsorption and covalent immobilization.

Based on above results, we deduced the configuration of each form of protein adsorption on the PS plate and on (PDDA/PAA)_3_ (Figure 8). On the PS plate, without OVA adsorption, the nonspecific adsorption of the secondary antibody clearly increased, because the plate did not have the protecting cover from the hydrophilic OVA; this resulted in a decrease in specific signal and an increase in noise. In contrast, on the (PDDA/PAA)_3_ substrate, even though the amount of physical adsorption of the primary antibody was lower than on the PS plate, the hydrophilic nature of the surface provided excellent performance for nonspecific protein adsorption inhibition; hence, the (PDDA/PAA)_3_ substrate provided a high-sensitivity ELISA system, with and without OVA adsorption. Moreover, the (PDDA/PAA)_3_ substrate could covalently immobilize the primary antibody to enhance its quantity, further increasing the antigen binding efficiency. The (PDDA/PAA)_3_ substrate system thus achieved the two essential factors of specific signal enhancement and noise reduction, significantly improving the conventional ELISA system, with simple processes and high sensitivity.

## 4. Conclusions

In a conventional ELISA system, we demonstrated that a (PDDA/PAA)_3_ substrate could directly inhibit nonspecific protein adsorption due to the hydrophilic nature of the outermost layer of PAA; high detection sensitivity was displayed, even though the blocking reagent adsorption step was omitted. Moreover, the (PDDA/PAA)_3_ substrate enriched the primary antibody via covalent immobilization to enhance the antigen binding efficiency, leading to a high specific signal and low noise in a conventional ELISA system with a simplified process. PEMs with an outermost layer of hydrophilic PAA could be used to modify a given substrate to create convenient and sensitive detection devices, improving conventional ELISA systems.

## Figures and Tables

**Table 1:** Results of the primary antibody and OVA adsorption.

Surface IgG ^a^	Primary (μg/cm^2^)	IgG Coverage ^b^ (%)		OVA ^a^ (μg/cm^2^)	OVA Coverage ^c^ (%)		Ratio of Molecules ^d^ (OVA/IgG)
		End-on	Side-on		End-on	Side-on	
PS	0.17 ± 0.01	9	63	0.72 ± 0.01	171	266	14.1
(PDDA/PAA)_3_	0.10 ± 0.01	5	36	0.21 ± 0.01	50	77	7.0
(PDDA/PAA)_3_PDDA	0.40 ± 0.01	21	146	1.91 ± 0.1	455	707	15.9

^a^ The dimension was calculated from the apparent area of filter (the diameter was 13 mm) (*n* = 3). ^b^ The dimensions of the IgG are 14.5 nm × 8.5 nm × 4 nm [33]. When one assumes full monolayer coverage, the amount of IgG adsorption was 1.85 and 0.27 μg/cm^2^ in the end-on and side-on positions, respectively. ^c^ The dimensions of the OVA are 7 nm × 5 nm × 4.5 nm [34]. When one assumes full monolayer coverage, the amount of OVA adsorption was 0.42 and 0.27 μg/cm^2^ in the end-on and side-on positions, respectively. ^d^ The molecular weights of IgG and OVA were 150 and 45 kDa, respectively.

**Table 2:** Amount of the primary antibody immobilization on the (PDDA/PAA)_3_.

Concentration of antibody (μg/mL)	Primary IgG ^a^ (μg/cm^2^)	IgG coverage ^b^ (%)	
		End-on	Side-on
10	0.28 ± 0.01	15	104
20	0.42 ± 0.01	23	156
30	0.56 ± 0.03	30	207

^a^ The dimension was calculated from the apparent area of filter (diameter was 13 mm). (*n* = 3); ^b^ The dimensions of the IgG are 14.5 nm × 8.5 nm × 4 nm [33]. When one assumes full monolayer coverage, the amount of IgG adsorption was 1.85 and 0.27 μg/cm^2^ in the end-on and side-on positions, respectively.

## Supplementary Materials

The following are available online at www.mdpi.com/2073-4360/9/2/51/s1, Figure S1: Amount of the primary antibody adsorption on the (PDDA/PAA)_3_ substrate by physical adsorption, Figure S2: S/N ratio of the antigen detection on the PS plate; (PDDA/PAA)_3_; and (PDDA/PAA)_3_PDDA substrate with OVA; and without OVA adsorption, Figure S3: Assembly of PDDA/PAA PEM on QCM substrate. The frequency shift and its thickness are indicated.
