# Supplementary material for: Hydrophilic Polyelectrolyte Multilayers Improve the ELISA System: Antibody Enrichment and Blocking Free
Source: Polymers (Basel). 2017 Feb 12;9(2):51. doi: 10.3390/polym9020051 (PMC6432497; doi:10.3390/polym9020051)
Supplement: Supplementary file 1 [file polymers-09-00051-s001.pdf]

Supplementary Materials: Hydrophilic Polyelectrolyte Multilayers Improve the ELISA System: Antibody Enrichment and Blocking Free

Xing Lai, Gan Gao, Junji Watanabe, Huiyu Liu and Heyun Shen

1. Determination the Amount of Physical Adsorption of Primary Antibody with Different Concentrations

An optimal concentration of primary antibody is essential for the ELISA system regardless of the immobilization method of the primary antibody being physical adsorption or covalent immobilization. The effect of the concentration of the primary antibody on the adsorption capacity was investigated. (Figure S1).

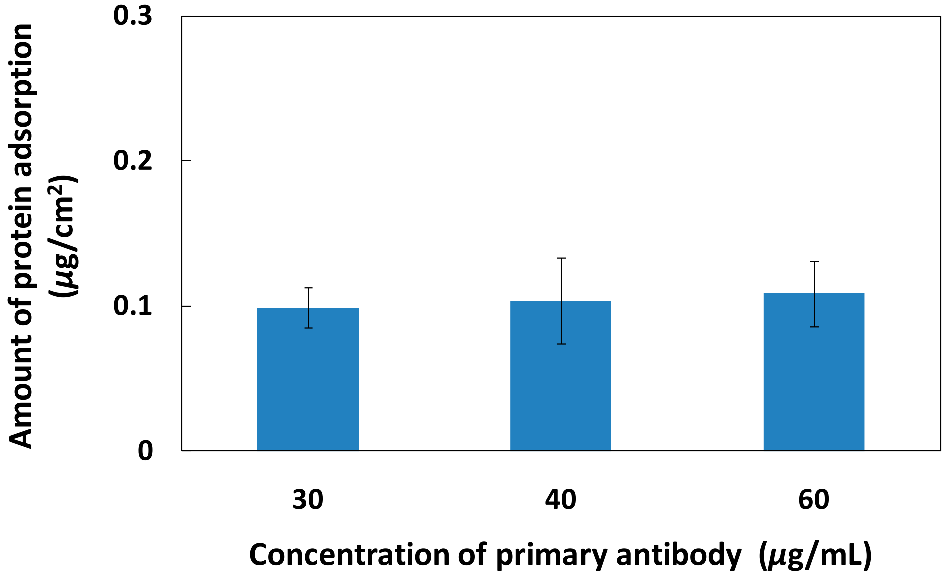

Figure S1. Amount of the primary antibody adsorption on the (PDDA/PAA)$_3$ substrate by physical adsorption at room temperature for 2 h (n = 3).

2. Optimal Conditions for Antigen Detection on Different Substrate

The secondary antibody must have an optimal concentration on each substrate for a higher sensitivity of the ELISA system. We confirmed the optimal concentration of the secondary antibody by the signal-to-noise ratio (S/N) of the antigen detection on the PS plate, (PDDA/PAA)$_3$, and (PDDA/PAA)$_3$PDDA substrate with OVA and without OVA adsorption (Figure S2).

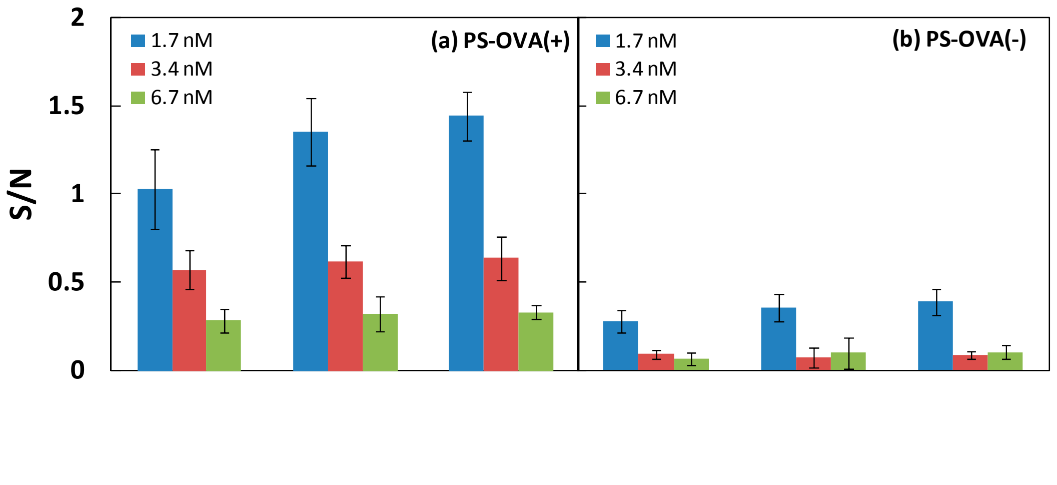

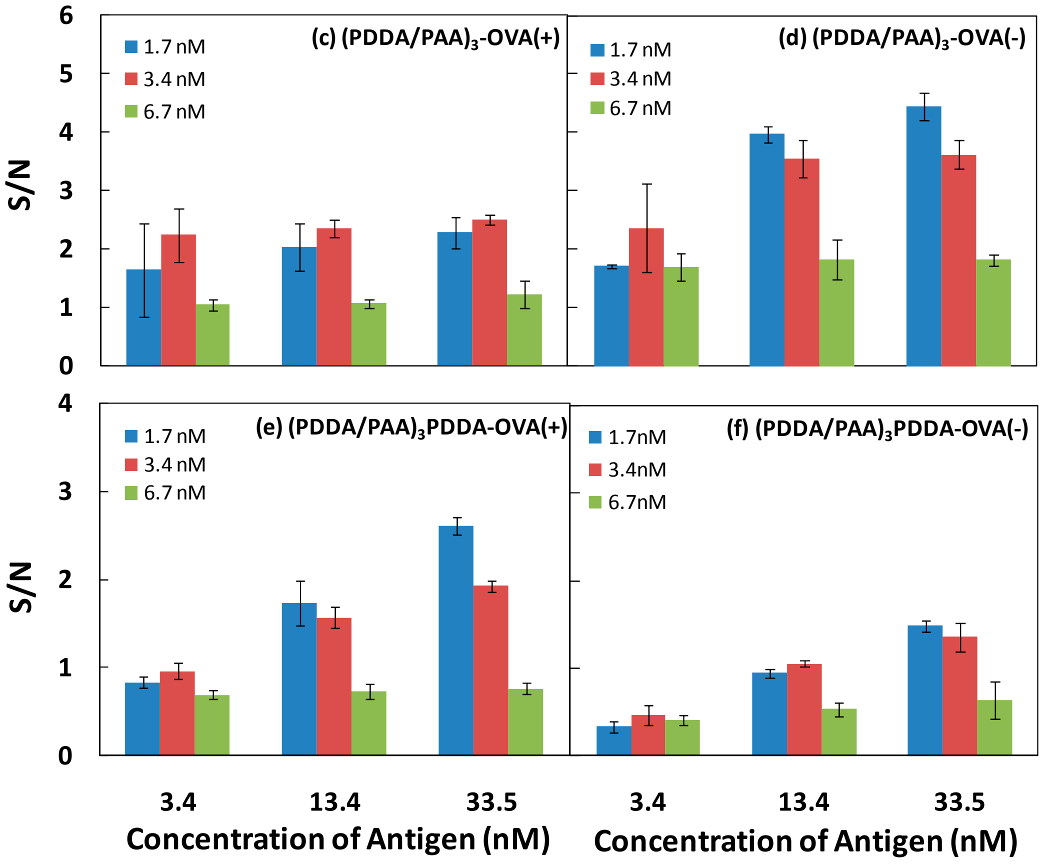

Figure S2. S/N ratio of the antigen detection on the PS plate (**a**,**b**); $(PDDA/PAA)_3$ (**c**,**d**); and $(PDDA/PAA)_3PDDA$ substrate (**e**,**f**) with OVA (**a**,**c**,**e**); and without OVA (**b**,**d**,**f**) adsorption (*n* = 3).

3. Investigation of PDDA/PAA PEM Formation on QCM Substrate

Quartz crystal microbalance (QCM) substrates were used for the preparation of the polyelectrolyte multi-layers. The most favorable characteristic of the QCM substrate was the ability to detect the instantaneous total weight of the polymer assembly at any time. Furthermore, the change in the frequency shift was correlated to the weight of the multi-layer using Sauerbrey's equation (1 Hz corresponds to 1.15 ng in the present study), and the detection limit was roughly 1 ng [1]. The QCM substrate used in the present study was an AT-cut quartz crystal with a parent frequency of 9 MHz, which was purchased from USI Co., Ltd, Fukuoka, Japan. The crystal (9 mm in diameter) was coated on both sides with gold electrodes 4.5 mm in diameter. The frequency was then monitored by a frequency counter (53131A, Agilent Technologies, Santa Clara, CA, US). The leads of the QCM were sealed and protected with a silicone-rubber gel in order to protect it from degradation during immersion in the aqueous solutions. The amount of polymer adsorbed was calculated by measuring the frequency decrease in the QCM, F, using Sauerbrey's equation as follows:

$$-\Delta F = \frac{2F_0^{\ 2}}{A(\rho_q \mu_q)^{1/2}} \Delta m$$

where F_0 is the parent frequency of the QCM (9 × 106 Hz), *A* is the electrode area (0.159 cm^2), q is the density of quartz (2.65 g/cm^3), and *q* is the shear modulus (2.95 × 1011 dyn/cm^2). This equation is reliable when the measurements are performed in air, as described in this study. The mass of the solvents was never detected as a frequency shift, and thus the effects of the viscosity of the absorbent can be ignored.

PDDA (M_w = 240,000 g/mol) was purchased from Polyscience Inc. PA, USA and PAA (M_w = 250,000 g/mol) was purchased from Wako Pure Chemical Industries, Ltd., Osaka, Japan. The polyelectrolyte solutions, PDDA and PAA, were dissolved in 50 mmol/L Tris-HCl (pH 7.4), and the concentration was adjusted to 0.2 mg/mL. The ionic strength was selected at 0 or 0.15 mol/L by using NaCl. Before polymer assembly, the QCM substrates on both sides were treated three times with a piranha solution (concentrated H_2SO_4/H_2O_2 (30 wt % aqueous solution) = 3/1, *v*/*v*) for 1 min, followed by rinsing with ultrapure water. After drying with N_2 gas, the cleaned QCM substrate was used for the preparation of the polyelectrolyte multi-layers.

The bare QCM substrate was immersed in a PDDA aqueous solution (0.2 mg/mL) for 1 min at a given temperature, and the substrate was then rinsed with 1 mmol/L Tris-HCl buffer solution to remove any surplus PDDA solution. After drying with N_2 gas, the frequency on the QCM was monitored. Next, the QCM substrate was immersed into a PAA aqueous solution (0.2 mg/mL), followed by rinsing with ultrapure water. After drying with N_2 gas, and the frequency monitoring was performed similarly. The alternate adsorption process was then repeated for the preparation of the polyelectrolyte multi-layers. NaCl was added at the given concentration into the each polymer aqueous solution, thus changing the polymer conformation. The apparent film thickness of the resulting assemblies was estimated from the frequency shifts. (Figure S3).

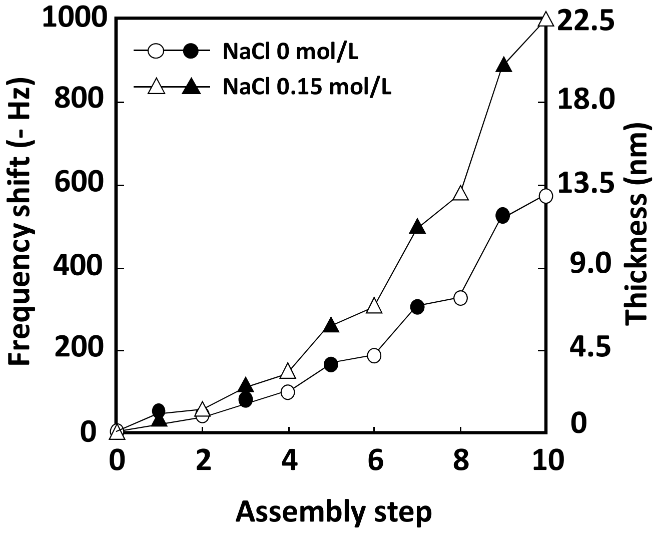

Figure S3. Assembly of PDDA/PAA PEM on QCM substrate. The frequency shift and its thickness are indicated. Polymer concentration was 0.2 mg/mL in 50 mmol/L Tris-HCl (pH 7.4). The ionic strength was adjusted to 0 or 0.15 mol/L by NaCl. PDDA was coated in the odd number steps, and PAA were coated in the even number steps (n = 2).

4. Conventional ELISA Protocol (Sandwich Assay)

The primary antibody (100 L/well) was adsorbed onto a 96-well microplate at 4 °C overnight to facilitate antigen detection. Next, bovine serum albumin (BSA) or OVA (1 mg/mL; 200 L/well) as blocking reagent was adsorbed onto each substrate at 37 °C for 1 h for inhibiting nonspecific protein adsorption (antigen or secondary antibody). Subsequently, the antigen-primary antibody, secondary antibody-antigen reactions were carried out at 37 °C for 1 h (100 L/well), respectively. Finally, the enzyme linked on the secondary antibody reacting with that on the substrate induced a color change to antigen quantification. The absorbance of the solution is measured by the multi-well plate reader. The microplate should be sufficiently rinsed after protein adsorption at every step.

Reference

1. Sauerbrey, G. Z. Verwendung von Schwingquarzen zur Wägung dünner Schichten und zur Mikrowägung. *Phys.* **1959**, *155*, 206.
